# Modelling of inactivation kinetics of *Escherichia coli*, *Salmonella* Enteritidis and *Bacillus subtilis* treated with a multi-hollow surface dielectric barrier discharge plasma

**DOI:** 10.1038/s41598-023-38892-2

**Published:** 2023-07-25

**Authors:** Silvia Mošovská, Veronika Medvecká, Ľubomír Valík, Anna Mikulajová, Anna Zahoranová

**Affiliations:** 1 Department of Nutrition and Food Quality Assessment, Faculty of Chemical and Food Technology, Slovak University of Technology, Radlinského 9, Bratislava, 811 07 Slovak Republic; 2 Department of Experimental Physics, Faculty of Mathematics, Physics and Informatics, Mlynská Dolina F1, Bratislava, 842 48 Slovak Republic

**Keywords:** Biophysics, Microbiology, Plasma physics

## Abstract

The efficacy of multi-hollow surface dielectric barrier discharge treatment against *Escherichia coli*, *Salmonella* Enteritidis and *Bacillus subtilis* was studied. Ambient air, O_2,_ and N_2_ were used as working gas with a flow rate of 6 l/m. Power delivered into plasma was 30 W over an area of 2 × 2 cm^2^. The active species in plasma generated in different gases participating in the inactivation of microorganisms were evaluated by optical emission spectroscopy and Fourier transform infrared spectroscopy. Inactivation curves were fitted to the Bigelow log-linear, the biphasic, and Geeraerd models. According to the results, all plasma treatments inactivated tested microorganisms, depending on a working gas. The most sensitivity of bacteria was observed to the ambient air plasma. Inactivation up to 5 log for *E. coli* and *S.* Enteritidis could be achieved within 15 s of plasma treatment. Air plasma exposure of 25 s also led to log_10_ CFU/ml of *B. subtilis* from 7.98 to 4.39. *S.* Enteritidis was slight resistance to plasma treatment with N_2_. Within 180 s nitrogen plasma treatment, a 2.04 log_10_ CFU/ml reduction was recorded.

## Introduction

The low temperature plasma (LTP) generated by various dielectric barrier discharges (DBDs) has displayed potential antimicrobial effect in many food matrices, including fresh produces, such as cherry tomatoes and strawberries^1^, spices^2–4^ or nuts^5,6^. The antimicrobial effects of LTP result from various possible reactions between different species that occur during plasma treatment^7^. The type and concentration of these reactive species depend on the plasma system, and the applied operating parameters, including working gas, moisture, and energy input^5–7^. For instance, reactive oxygen (hydrogen peroxide, hydroxyl radical, superoxide, singlet oxygen, atomic oxygen and ozone) and nitrogen species (peroxynitrite, nitric oxide and nitrite), UV photons, and charged particles are the essential bactericidal agents in LTP generated in ambient air^7,8^.

The OH radical, a ROS formed in plasma, plays a significant role in inactivation of various pathogens as a consequence of its high oxidation potential^9^. According to Procházka et al.^10^, a coplanar DBD ignited in water vapour enhances the generation of OH radicals. However, the addition of water vapour to the air leads to the increase of voltage required for LTP generation or even prevent plasma generation. Mentioned limitations lead to the development of the effective discharge design for the reliability of the plasma source in a potential application^9^.

A new geometry of multi-hollow surface dielectric barrier discharge (MSDBD) combines the surface and volume geometry of DBD plasma systems^11,12^. The MSDBD consists of two parallel electrodes fully embedded in ceramics to prevent erosion of electrodes. The MSDBD system contains 105 holes, inside which the plasma is generated in appropriate working gas, and the flow (5–20 L/min) ensures the transfer of active particles to the treated sample. In addition, the unique geometry and cooling effect lead to a high yield of active particles, including ozone, and enable the plasma treatment of samples at higher distances or models with a structured surface^11^. The detailed description of MSDBD geometry and properties of generated plasma can be found in paper of Homola et al.^13^.

Microbial food safety and preservation techniques are one of the most critical issues in the food industry. Foodborne infections caused by pathogenic microorganisms may negatively influence public health and socio-economic development^14^. The food contamination with spoilage bacteria may lead to the presence of microbial toxin and thus it represents a health hazard to the consumers^1^. For instance, an outbreak of Shiga-toxin-producing *Escherichia coli* O104: H4 was linked to the consumption of fenugreek sprouts in Germany in 2011^15^. In addition, pathogens, such as *E. coli* O157:H7 and *Salmonella* spp. may be able to survive for long periods of time^1^. On the other hand, *Bacillus subtilis*, one of the most frequently occurred spore-forming bacteria in spices, is a food-poisoning bacteria. This bacterium is known as a human non-pathogen but it occasionally causes characteristic toxi-infections leading to severe vomiting, abdominal cramps and diarrhoea. In addition, *B. subtilis* is able to survive the sterilization process^16^.

As indicated above, many studies have focused on the inactivation of pathogenic microorganisms by LTP. However, utilizing ambient air presents numerous challenges in the process of generating plasma (excessively demanding power requirements, elevated gas temperatures, and disruptive instabilities)^13^ what leads to the development of alternative design of the discharge configurations. Various studies showed the potential of MSDBD to become.practical for plasma treatment in food processing^17,18^. The study of Kelar Tučeková et al.^9^ showed the potential of MSDBD treatment to decontaminate bacterial biofilm.

However, to our knowledge, no studies describe the antimicrobial effects of MSDBD treatment for the decontamination of pathogenic bacteria contaminated food. In addition, modelling inactivation kinetics could provide important information about the mechanism of plasma treatment. Inactivation kinetics can be useful tool to predict and compare the behaviours of inactivation of microorganisms at specific processing conditions^19^. Furthermore, it can predict the best range of treatment conditions for the most effective inactivation. In case of minimal processing, nonlinear characteristics such as tailing, shoulder, and sigmoid effects are usually observed in inactivation. These nonlinearities could be well described by various widely used non-linear kinetic models^19,20^, including Weibull^21^, the Biphasic model^22^ and many more.

Therefore, the aim of the study was to determine and compare the kinetic behaviour of *E. coli*, *S.* Enteritidis, and *B. subtilis* treated with MSDBD plasma generated in different working gases. The kinetic parameters for bacterial cell inactivation were also estimated. Furthermore, optical emission spectroscopy and Fourier transform infrared spectroscopy were used to characterise the various generated plasmas.

## Material and methods

### Bacterial strains

The tested bacterial strains, *E. coli* CCM 3988, *S. enterica* subsp. e*nterica* serovar Enteritidis CCM 4420, *B. subtilis* CCM 1999 (vegetative form), were obtained from the Collection of Microorganisms, Masaryk University, Brno, Czech Republic.

The bacterial strain was grown in Nutrition Agar at 37 °C for 24 h and then kept at 4 °C. Before each assay, 7 ml of sterile nutrition broth was aseptically inoculated with a colony of the tested bacteria. The cell suspension was incubated for 16 h at 37 °C under continuous shaking (320 rpm). Approximate viable cell density was 2–5 × 10^8^ cells/ml.

### Sample preparation

Melted sterile Nutrition agar No. 2 was transferred to a thermostat and kept at 60 °C for 30 min. Subsequently, 2.5 ml of prepared nutrition agar was pipetted into a sterile glass plate. After solidifying agar (at 25 °C), the disk was cut with a sterile cutting device (Ø 22 mm) and carefully transferred into a sterile slide placed in a Petri dish. The Petri dish content was allowed to dry overnight at room temperature. Following 15 µl of the obtained bacterial cell suspension (tenfold diluted) was spread on a disk and allowed to soak up for about 1 h at room temperature.

### Plasma source and plasma treatment

**Figure 1:**
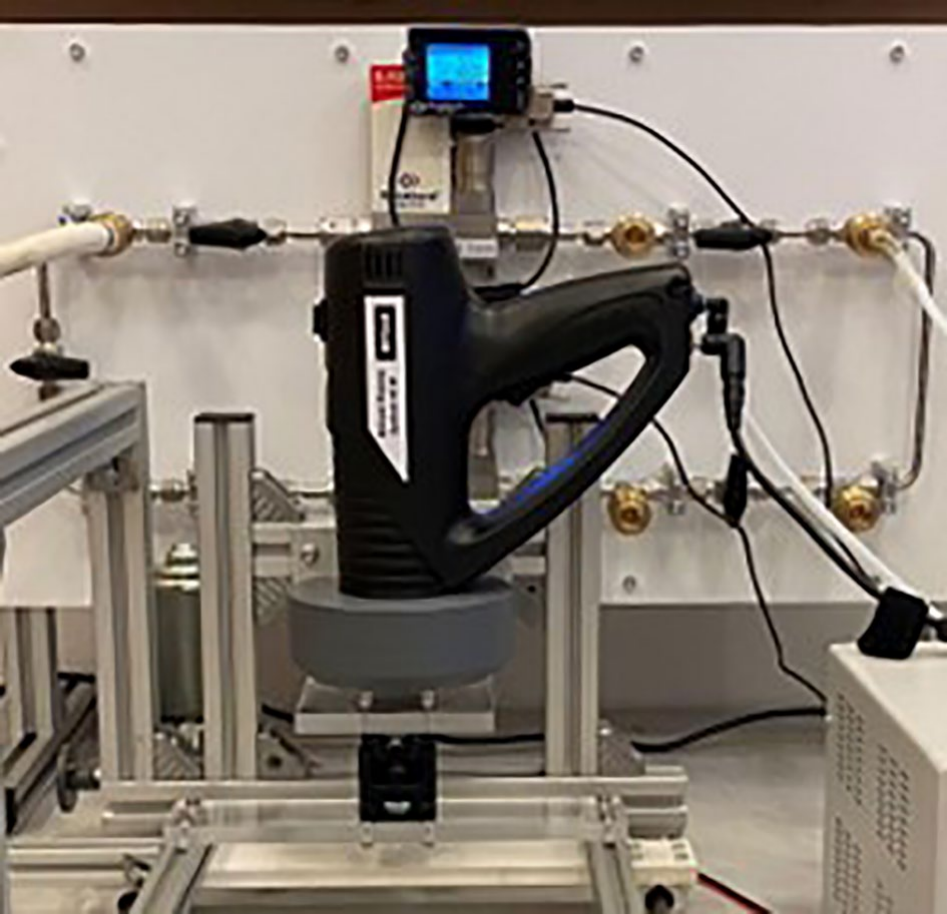
The MSDBD apparatus.

The plasma used (Fig. 1) for the treatment of samples was generated by the RPS30 device (Roplass Ltd, CZ) with a multi-hollow surface dielectric barrier discharge (MSDBD) plasma unit^12^. The conditions were optimized based on the previous research^13^. The working gas (ambient air, oxygen and nitrogen) flows through an array of 105 hollows in the MSDBD system with a flow rate of 6 l/m. The area of plasma is 2 × 2 cm^2^, and the power delivered into plasma was 30 W. The samples were treated at distance of 1 mm from the surface of MSDBD ceramics. The plasma treatment was carried out in intervals of 45 s. After that, the RPS30 was cooled for 200 s. In exposure time of less than 45 s, the cooling time was proportional to exposure time to maintain the stable temperature during plasma treatment.

The samples were treated up to 180 s. The total treatment time varied according to working gas and used bacteria strains. After the treatment, the reactor was cleaned immediately using isopropyl alcohol and dried with the working gas. The experiments were performed in triplicate.

### Optical emission spectroscopy and Fourier transform infrared spectroscopy

The composition of plasma generated by MSDBD was analysed by optical emission spectroscopy (OES) and Fourier transform infrared (FTIR) spectroscopy.

The emission spectra of plasma generated in ambient air, oxygen and nitrogen were acquired in a dynamic regime by spectrometer StellarNet EP-2000 in the range of 200–1100 nm (StellarNet, USA). The detector integration time was 100 ms, and the spectra were integrated during 100 scans. The optic fibre was placed on an axis perpendicular to the plane of the ceramics at a distance of 5 cm. The FTIR in ambient air, O_2_, and N_2_ were measured by spectrometer Bruker Vector 22 (Bruker Optics, USA) in a spectral range of 4000–500 cm^−1^ with the resolution of 4 cm^−1^ with 8/8 scans for background (working gas) and plasma switched on. Spectra were acquired in the flow regime, and the products generated in plasma were led into the cuvette with a path length of 10 cm.

### Microbial analysis of bacterial cell recovery

The number of survived bacteria after MSDBD treatment was evaluated by the standard plate count method. The plasma treatment disk was immersed into 3 ml of 0.85% sterile saline solution; the mixture was vortexed for 60 s at room temperature. The obtained suspension was serially diluted, and every solution was placed on Mueller–Hinton agar plates. The plates were incubated for 24 h at 37 °C, and colony-forming units (CFU) were counted. Non-treated agar ring was used as a control sample.

### Determination of inactivation kinetic parameters

Inactivation curves were constructed by plotting log survivor fraction against plasma treatment time. The kinetic parameters for bacterial cell inactivation were estimated using log-linear, biphasic, and Geeraerd models.

Log-linear model^23^ was fitted to the inactivation data using GInaFit software (version 1.7 for Microsoft Excel^24^) as is shown in Eqs. (1 and 2):

1 $${\text{log}}N_{t} = {\text{ log}}N_{0} {-}{\text{ k}}_{{{\text{max}}}} *{\text{t}}/{\text{ln}}_{{{1}0}}$$

2 $${\text{log}}N_{t} = {\text{ log}}N_{0} {-}{\text{ t}}/D$$

where *N*_*t*_ represents the counts of surviving viable cell (CFU/ml) after plasma treatment at the time t; *N*_*0*_ represents the initial cell populations (CFU/ml) at the time 0; *D* is the decimal reduction time (*D*-value); k_max_ represents the specific inactivation rate (s^−1^); t represents the exposure time of each treatment (s).

The biphasic model^22^ was modelled using GInaFit software (version 1.7 for Microsoft Excel^24^) as is shown in Eqs. (3–5):

3 $${\text{log}}N_{t} = {\text{log}}N_{0} + {\text{ log }}\left( {{\text{f}}*{\text{exp}}\left( { - {\text{k}}_{{{\text{max1}}}} *{\text{t}}} \right) + \, \left( {{1} - {\text{f}}} \right)*\left( { - {\text{k}}_{{{\text{max2}}}} *{\text{t}}} \right)} \right)$$

4 $$D_{1} = {1}/{\text{k}}_{{{\text{max1}}}}$$

5 $$D_{2} = {1}/{\text{k}}_{{{\text{max2}}}}$$

where *Nt* represents the counts of surviving viable cell (CFU/ml) after plasma treatment at the time t; *N*_*0*_ represents the calculated initial cell populations (CFU/ml); k_max1_ (s^−1^) and k_max2_ (s^−1^) represent the rates of inactivation for the population corresponding to the subpopulation more sensitive to the treatment (f) and the population corresponding to the subpopulation more resistant to the treatment (1 − f), respectively; *D*_*1*_- and *D*_*2*_- is *D*- value for both regions.

The Geeraerd model^25^ was modelled using GInaFit software (version 1.7 for Microsoft Excel^24^) as is shown in Eq. (6):

6 $${\text{log}}N_{t} = {\text{log }}\left( {{1}0^{{{\text{log}}N0}} - { 1}0^{{{\text{log}}Nres}} } \right)*{\text{exp}}^{{ - kmax*{\text{t}}}} + { 1}0^{{{\text{log}}Nres}}$$

7 $$D = {1}/{\text{k}}_{{{\text{max}}}}$$

where *Nt* is the surviving microbial count (CFU/ml) after plasma treatment at the time t; *N*_*0*_ is the calculated initial cell populations (CFU/ml); *N*_*res*_ is the residual subpopulation (CFU/ml); k_max_ (s^−1^) is the maximum specific inactivation rate; *D* is decimal reduction time (s)

### Statistical analysis

Replicate data sets were plotted to obtain mean survivor curves together with standard deviations. Significant differences between means were determined using one-way analysis ANOVA followed by Bonferroni correction post hoc test at *p* ˂ 0.05 level.

The values represent the means ± standard deviation (SD) obtained from four experiments.

## Results

### Characterization of the plasma

**Figure 2:**
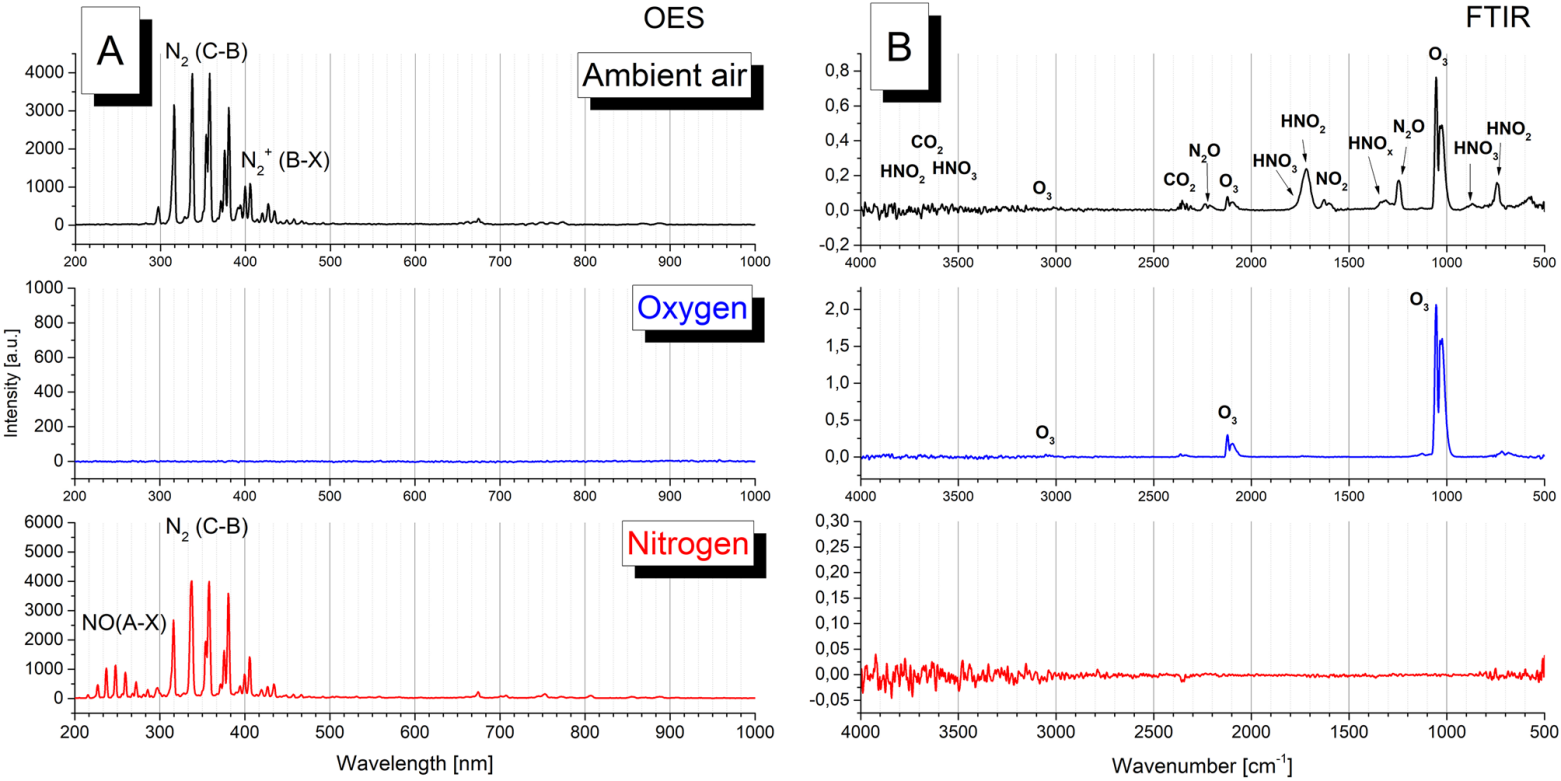
The optical emission spectra of plasma (**A**) and FTIR analysis of gaseous products (**B**) generated by MSDBD in different working gas (ambient air, oxygen, nitrogen).

Optical emission spectra of plasma generated by MSDBD in different working gases are present in Fig. 2 A. The dominant radiative transition visible in spectra acquired in ambient air and nitrogen plasma is second positive system of nitrogen molecule N_2_ (C^3^ Π_u_ → B^3^ Π_g_). This system is the primary radiation source in the UV region and takes place in decontamination effect of plasma in a nitrogen-containing atmosphere^26^. With low intensity, the first negative system of N^+^_2_ (B^2^ Σ^+^_u_ → X^2^ Σ^+^_g_) and first positive system N_2_ (B^3^ Π_g_ → A^3^ Σ^+^_u_) can be detected.

In oxygen, the emission spectra in the measured region did not show any radiative transitions. On the other hand, spectra acquired in nitrogen exhibit strong peaks attributed to the second positive system and also radiation of NO molecule (A^2^ Σ^+^ → X^2^ Π) by the interaction of ambient air with nitrogen species generated in working gas.

FTIR analysis (Fig. 2B) of gaseous products measured in ambient air showed a variety of active particles; the dominant peaks are attributed to ozone, nitrogen oxides (N_2_O, NO_2_) and HNO_2_. In addition, HNO_3_ were detected due to the presence of air humidity in ambient air. These species are powerfully bioactive and play an essential role in the decontamination effect of plasma. In oxygen, the intensive production of ozone was observed. The MSDBD, thanks to the configuration and operation in the flow of working gas, is a plasma source with a high ozone yield. In Homola et al.^11^, ozone production efficiency was found to be higher than other standard DBD geometries—coplanar, surface and volume DBDs.

**Figure 3:**
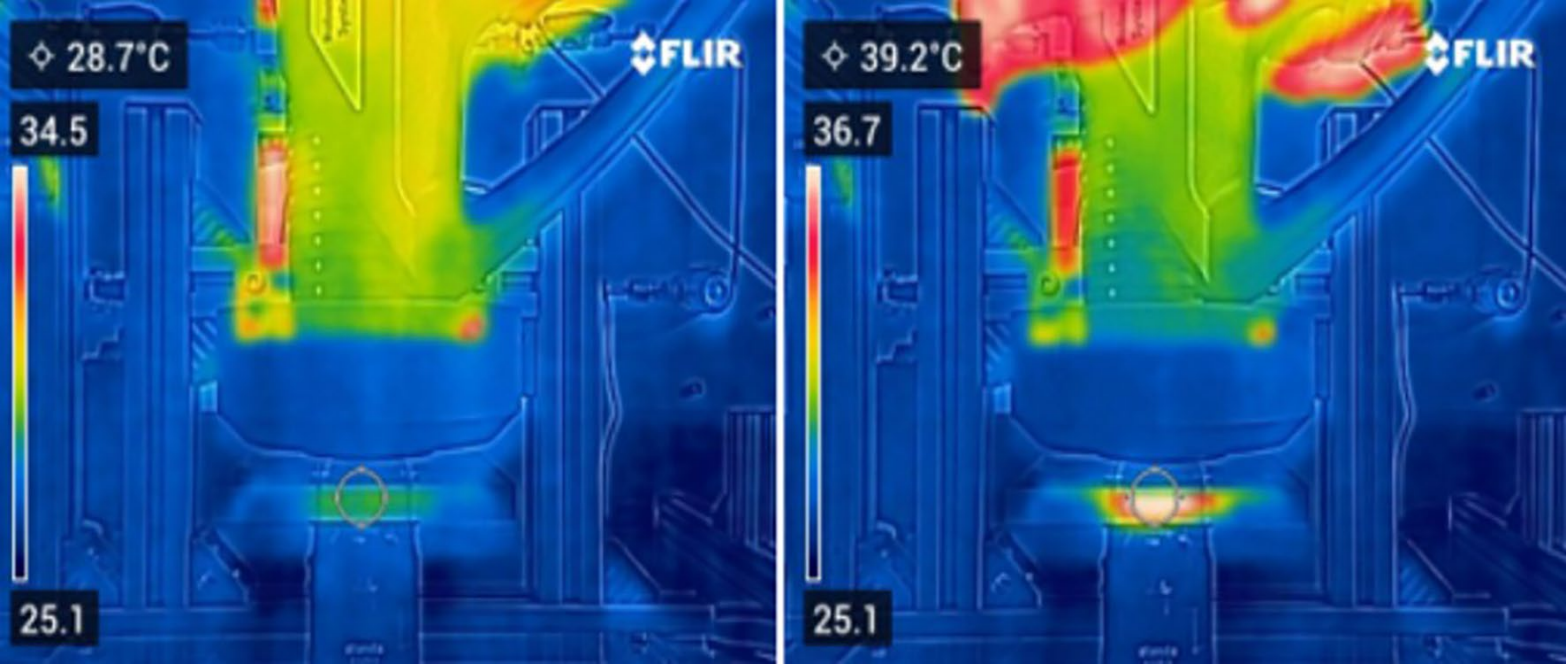
The picture of MSDBD using thermal camera at 0 s and 45 s; temperature measured in the treatment area between ceramics and table used for samples holding.

The temperature of the surrounding objects in contact with the plasma (especially the table used for sample holding and the ceramic surface) were monitored by the thermal camera Flir C5TM Compact Thermal Camera (Teledyne Flir, Estonia). The maximal temperature in the treatment area was up to 40 °C (Fig. 3). In addition, the flowing working gas has significant cooling effect^13^.

### Inactivation kinetics of bacteria under plasma treatments

**Figure 4:**
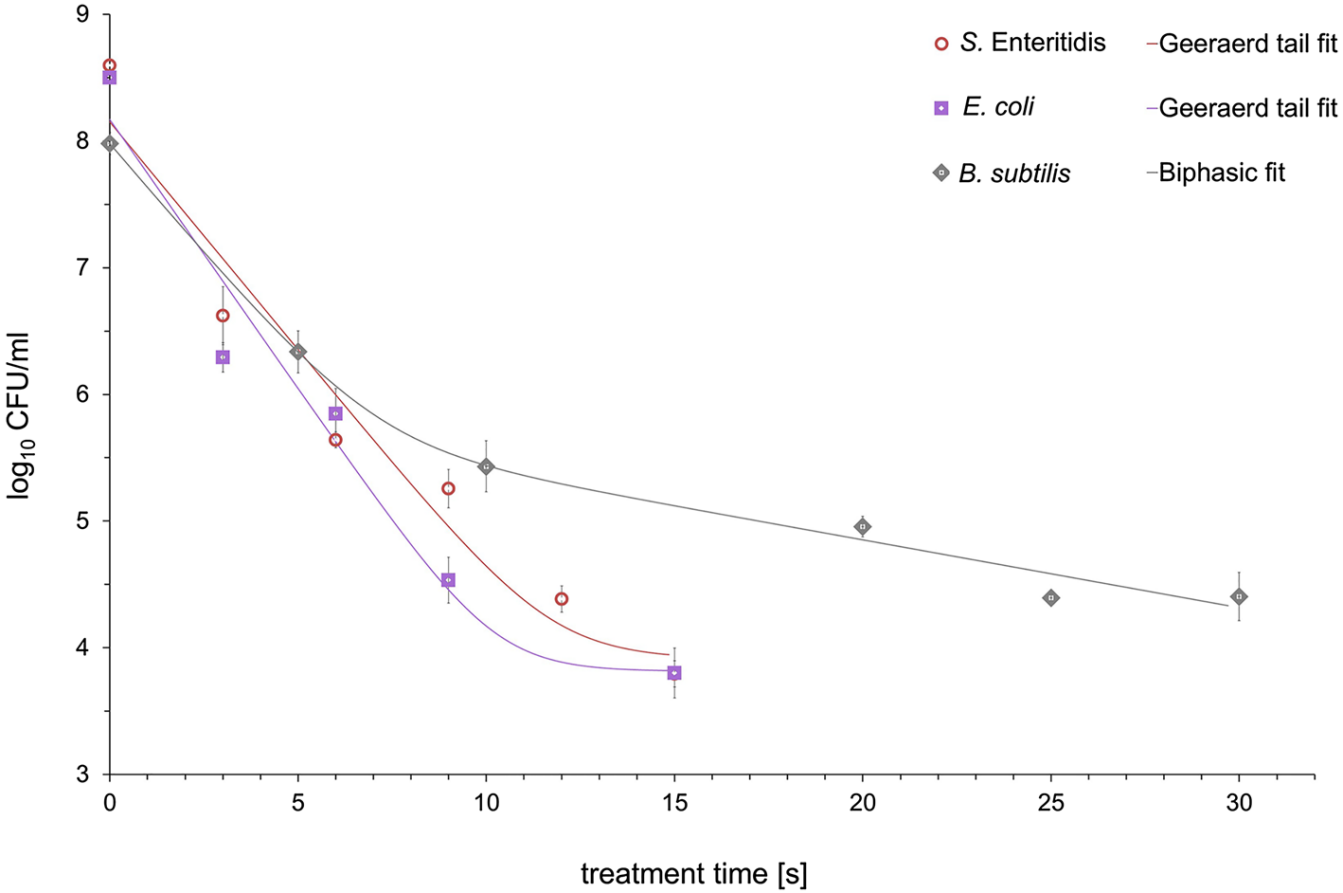
Effects of MSDBD plasma treatment on the inactivation of *E. coli*, *S.* Enteritidis, and *B. subtilis* for ambient air as a working gas.

**Figure 5:**
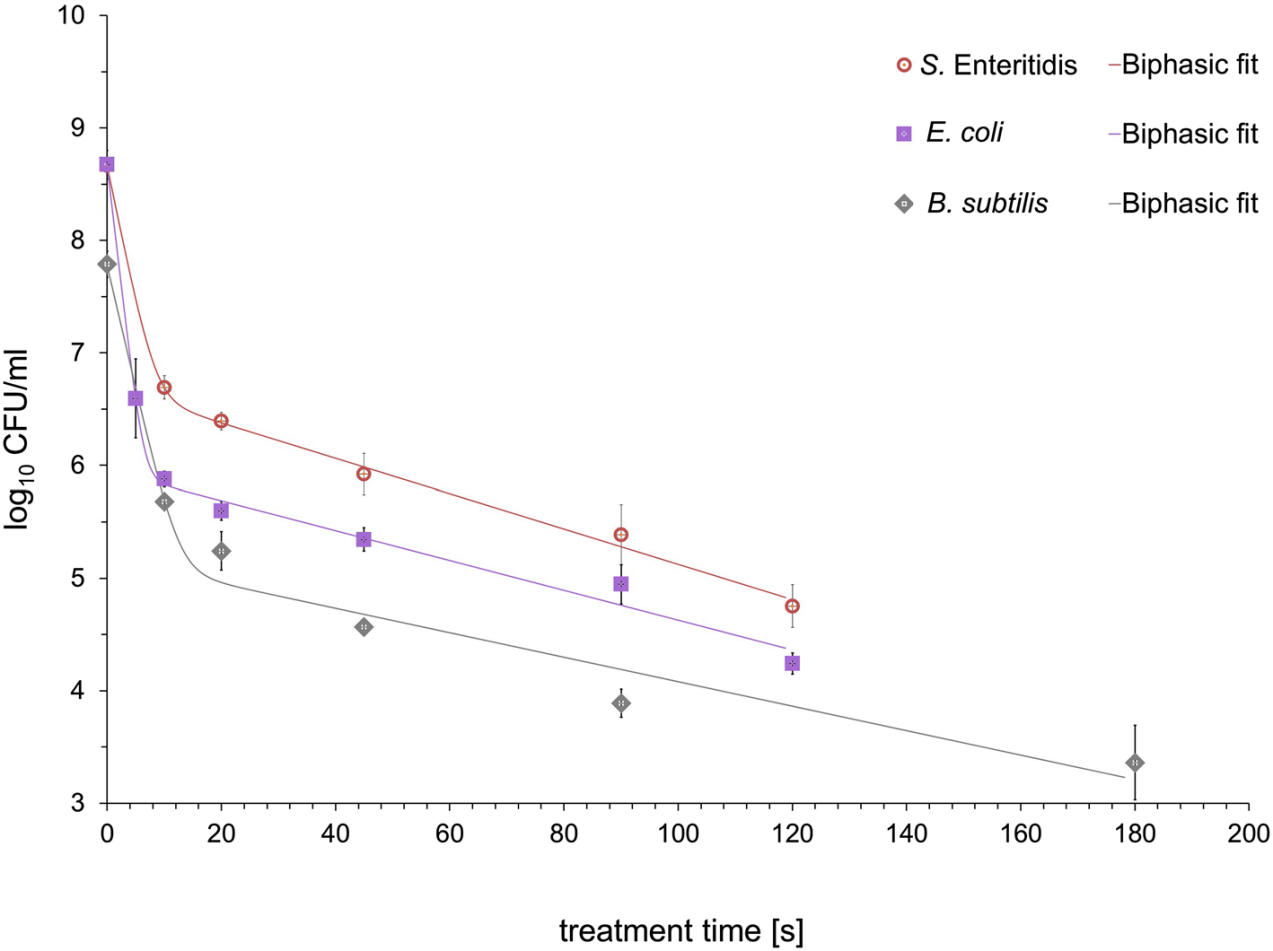
Effects of MSDBD plasma treatment on the inactivation of *E. coli*, *S.* Enteritidis, and *B. subtilis* for oxygen as a working gas.

**Figure 6:**
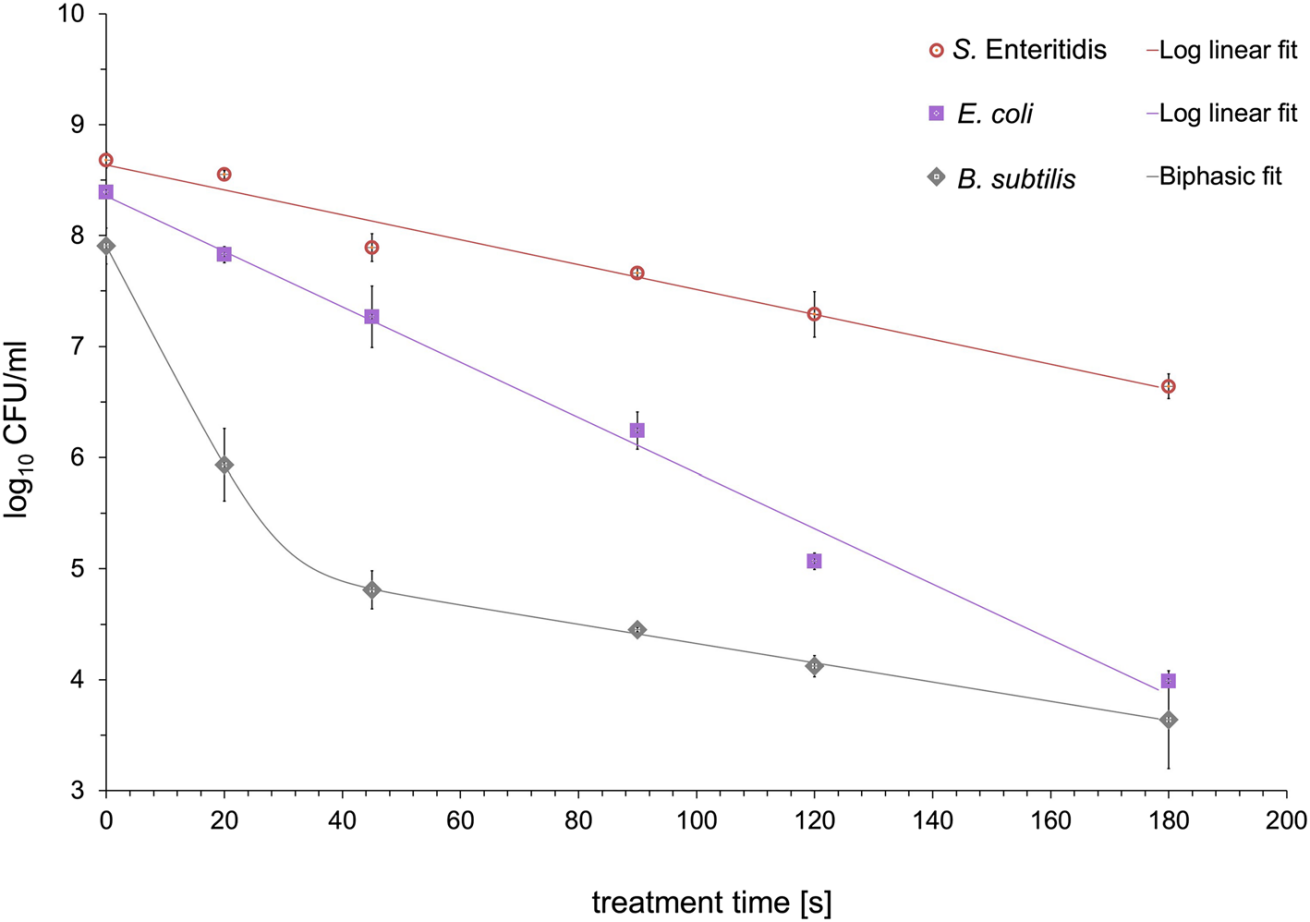
Effects of MSDBD plasma treatment on the inactivation of *E. coli*, *S.* Enteritidis, and *B. subtilis* for nitrogen as a working gas.

A time course study of the inactivation of *E. coli*, *S.* Enteritidis and *B. subtilis* by different plasmas clearly showed variation in sensitivity to this treatment for the three strains. The kinetic profiles of the survival curves of tested microorganisms obtained from various working gases, seemed to be non-linear on a semi-logarithmic scale (Figs. 4 and 5) with the exception of nitrogen plasma for *E. coli* and *S.* Enteritidis (Fig. 6). The fitted parameters of inactivation kinetics for all models for tested microorganisms treated by MSDBD plasma generated in different process gases are shown in Table 1. The results of the statistical analysis on kinetic changes for all tested microorganisms to the plasma treatment using predictive models are summarized in Table 2.

**Table 1:** The fitted parameters of inactivation kinetics for Geeraerd, biphasic, and log-linear models for tested microorganisms treated by MSDBD plasma generated in different process gases.

Process gasses	Parameters	*E. coli*	*S.* Enteritidis	*B. subtilis*
Ambient air	log N_0_ (CFU/ml)	8.2 ± 0.4	8.2 ± 0.4	8.0 ± 0.2
	Model	Geeraerd	Geeraerd	biphasic
	*k*_*max1*_ (s^−1^)	1.0 ± 0.2	0.8 ± 0.2	0.8 ± 0.1
	*k*_*max2*_ (s^−1^)	NA	NA	0.12 ± 0.03
	log N_res_ (CFU/ml)	3.8 ± 0.5	3.9 ± 0.5	NA
	*f*	NA	NA	0.991 (0.007)
	*D*_*1*_-value (s)	1.0 ± 0.2	1.2 ± 0.2	1.3 ± 0.2
	*D*_*2*_-value (s)	NA	NA	8 ± 2
O_2_	log N_0_ (CFU/ml)	8.7 ± 0.1	8.7 ± 0.1	7.8 ± 0.3
	Model	Biphasic	Biphasic	Biphasic
	*k*_*max1*_ (s^−1^)	1.0 ± 0.1	0.57 ± 0.09	0.5 ± 0.1
	*k*_*max2*_ (s^−1^)	0.031 ± 0.003	0.036 ± 0.003	0.025 ± 0.006
	*f*	0.998 (0.001)	0.989 (0.003)	0.998 (0.002)
	*D*_*1*_-value (s)	1.0 ± 0.1	1.8 ± 0.3	2.0 ± 0.5
	*D*_*2*_-value (s)	33 ± 4	28 ± 2	40 ± 10
N_2_	log N_0_ (CFU/ml)	8.4 ± 0.1	8.6 ± 0.1	7.91 ± 0.04
	Model	log-linear	log-linear	biphasic
	*k*_*max1*_ (s^−1^)	0.057 ± 0.003	0.026 ± 0.002	0.233 ± 0.006
	*k*_*max2*_ (s^−1^)	NA	NA	0.020 ± 0.001
	*f*	NA	NA	0.998 (0.001)
	*D*_*1*_-value (s)	17.4 ± 0.8	39 ± 3	4.3 ± 0.1
	*D*_*2*_-value (s)	–	–	50 ± 2

^NA^ not applicable; *value is calculated.

**Table 2:** Statistical parameters of Geeraerd, biphasic, and log-linear models for MSDBD plasma treatment generated by different process gases.

Process gasses	Parameters	*E. coli*	*S.* Enteritidis	*B. subtilis*
Ambient air	MSSE	0.263	0.228	0.027
	RMSSE	0.513	0.478	0.165
	R^2^	0.960	0.960	0.994
O_2_	MSSE	0.020	0.009	0.102
	RMSSE	0.140	0.097	0.319
	R^2^	0.995	0.998	0.983
N_2_	MSSE	0.031	0.020	0.001
	RMSSE	0.175	0.142	0.035
	R^2^	0.991	0.973	1.000

#### Ambient-air based plasma

The surviving population of *E. coli*, *S.* Enteritidis and *B. subtilis* after air based plasma treatment is presented in Fig. 4. The inactivation effect increased with exposure time; all tested bacteria showed non-linear inactivation behaviour (Table 1).

Due to its flexibility, the Weibull model was initially used. However, based on the graphical processing of the inactivation data using the Weibull model, a highly probable presence of subpopulations with different resistances was identified. Therefore, a biphasic Cerf model^22^ was applied. In our case, the advantage of this model was its ability to provide suitable parameters *k*_*max1*_ and *k*_*max2*_, *D*_*1*_*-* and *D*_*2*_*-* value, which have practical significance and ultimately confirmed the presence of two subpopulations of tested microorganisms with different sensitivities to plasma treatment. However, the Cerf model for *E. coli* and *S.* Enteritidis exhibited higher errors in model parameters (data not shown). This issue was resolved by fitting the experimental results with Geeraerd model^25^.

The data suggested that the models satisfactorily explains the inactivation patterns of tested microorganisms obtained from different plasmas; a R^2^ value ˃ 0.9 (Table 2). *E. coli* and *S.* Enteritidis appeared to be more sensitive to the ambient air-based plasma treatment (Fig. 4). The treatment for 15 s reduced population of *E. coli* and *S.* Enteritidis by 4.70 log_10_ and 4.81 log_10_, respectively. Compared to the initial population of *B. subtilis* (7.98 ± 0.087 log_10_ CFU/ml), the microbial reduction level of 3.59 log_10_ was reached in 25 s of sample exposure in plasma.

For kinetic analysis, the largest fraction of the initial population was inactivated during the first part of treatment. From the biphasic Cerf and the Geeraerd models, the inactivation rates (*k*_*max1*_) and decimal reduction time (*D*_*1*_) for sensitive subpopulations were similar among the tested bacteria (Table 1). For that reason, the antibacterial effect of ambient air plasma was comparable in the first phase of inactivation curve in the tested bacterial strain. In addition, the highest inactivation rates for sensitive subpopulations indicated the fastest inactivation of *S.* Enteritidis and *B. subtilis* achieving in air plasma compared with O_2_- and N_2_-based plasma treatment.

#### Oxygen based plasma

O_2_-based plasma treatment also resulted in non-linear inactivation behaviour in all tested bacteria (Fig. 5), indicating the presence of sensitive and a more resistant fractions within the initial population. However, according to *D* –values obtained from Cerf model, studied bacteria were slightly more resistant to O_2_-based plasma than the air plasma treatment (Table 1). *E. coli* presented lower *D*_*1*_-value of sensitive cells and higher inactivation rates for the sensitive fraction (*k*_*max1*_), indicating the highest sensitivity to the O_2_-based plasma treatment among tested bacteria. After 120 s of treatment, the initial population of *E. coli* (8.67 ± 0.128) were decreased by 4.43 log_10_. Compared to the initial population of *S.* Enteritidis (8.67 ± 0.020), the microbial reduction level of 3.92 log_10_ was achieved in 120 s of sample exposure in plasma. The data demonstrated that *B. subtilis* was more resistant with low inactivation rates and the highest *D*-values for the sensitive and resistant fraction (Table 1). Despite of slower inactivation kinetics, O_2_-based plasma treatment reduced the initial number of *B. subtilis* (7.79 ± 0.116) by 4.43 log_10_.

#### Nitrogen based plasma

MSDBD plasma treatment using N_2_ as a working gas resulted in a different inactivation behaviour of treated bacteria (Fig. 6). Our results suggested similarity of behaviour and ability to survive of nitrogen plasma-treated *B. subtilis* with oxygen plasma treatment (Table 1). For kinetic analysis, the inactivation curve of *B. subtilis* contained an upward concavity. The tailing-off of the inactivation curve indicates the presence of a sensitive and a more resistant fractions within the initial population. *B. subtilis* showed the highest level of *k*_*max1*_ and the lowest level of *D*_*1*_- value for the sensitive fraction, indicating the fastest kinetics of devitalisation among tested microorganisms. Subsequently, a CFU reduction of resistant fraction was mitigated. The inactivation curves of *E. coli* and *S.* Enteritidis obtained by N_2_-based plasma proceeded according to a linear pattern (Fig. 6), and the experimental data was fitted using a log-linear model^23^. High R^2^ and low RMSSE values showed the appropriate fitting ability of a particular model to describe the inactivation data for both pathogens (Table 2). Despite of slower inactivation kinetic for *E. coli,* the effect of nitrogen plasma on the mentioned bacteria was comparable with *B. subtilis*. Compared to the initial population of *B. subtilis* (7.91 ± 0.16 log10 CFU/g sample) and *E. coli* (8.39 ± 0.03 log10 CFU/g sample), the microbial reduction level of 4.27 log10 and 4.40 log10 was achieved in 180 s of samples exposure in plasma. On the other hand, *S.* Enteritidis showed the highest cell survival compared with *E. coli* and *B. subtilis*. Within 180 s of N_2_-based plasma treatment, a 2.04 log_10_ CFU reduction was observed. According to the data, *S.* Enteritidis was more resistant with low inactivation rate.

## Discussion

Mathematical modelling of microbial growth or inactivation kinetics is one of the essential elements of quantitative exposure and risk assessment. Furthermore, predictive modelling can be used to compare the effectiveness of different processing technologies in reducing microbial populations^27^.

To establish appropriate plasma treatment conditions, applying a reliable kinetic model for microbial inactivation is essential^19^.

In the present study, multi-hollow surface dielectric barrier discharge (MSDBD) has been successfully tested for inactivation of *E. coli*, *S.* Enteritidis and *B. subtilis*, depending on working gas.

Reactive species (ROS, RNS, ozone etc.) are considered to be a crucial factor responsible for the effect of low temperature plasma on living cells^28^. Depending on the plasma system and the applied operating parameters, complex mixture of different components are formed which can contribute to the inactivation of microorganisms^7,28^. As seen in Fig. 2 A and B, MSDBD generated in ambient air provides a wide variety of bioactive nitrogen and oxygen species. Compared to standard DBDs geometries (coplanar, surface or volume)^3^, MSDBD plasma generated in ambient air also exhibits significantly higher efficiency of ozone production due to the cooling effect of produced active species by a flow of working gas, the properties of ceramics and geometry of electrode system. In addition, the presence of water as air humidity leads to the formation of HNO_2_ and HNO_3_ from nitrogen oxides and hydroxyl radicals, one of the most active ROS. The coplanar DBD ignited in water vapour generates plasma with 10 times higher concentration of OH radicals than the discharge in humid air^10^. According to Eto et al.^29^, OH radicals might be produced by the chemical reaction between ozone and water vapour and play a role in the inactivation of *Geobacillus stearothermophilus* spores. Furthermore, the generated air plasma was a source of nitrogen species and UV photons (Fig. 2A).

Inactivation curves of *E. coli*, *S.* Enteritidis and *B. subtilis* under various plasmas showed significant tailing (Fig. 4 and 5) with the exception of nitrogen plasma for *E. coli* and *S.* Enteritidis (Fig. 6). The measured inactivation data were fitted with three first-order kinetic models, including the Bigelow-log linear^23^, the biphasic Cerf^22^ and Geeraerd^25^ models. The biphasic model is usually used for inactivation curves in consequence of a non-zero activity during prolonged treatments^19,27^. On the other hand, the Geeraerd model describes the sensitive and the resistant population, which is not inactivated^25^. R^2^ (˃ 0.9) indicated the suitability of used models for describing the inactivation kinetics (Table 2). The results suggested that pathogens were more susceptible to ambient-air plasma exposure since their initial counts were reduced considerably after shorter treatment times compared with O_2_- and N_2_-based plasmas. During air- and O_2_-based plasmas, the inactivation behaviours of tested bacteria were similar in the shape of the curves; a quicker inactivation rate at the beginning of the plasma treatment was followed by tailing (Figs. 4 and 5). The shape of the inactivation data suggested the presence of a plasma sensitive and a plasma resistant population (Table 1). Except for the different inactivation mechanisms involved during the treatment^4^, the occurrence of tailing could also be explained by the physicochemical processes proceed during inactivation. Inactive cells could create a mechanical barrier and could be a preferred target for active particles. The degree of saturation of active particles should also be taken into account, as well as repair mechanisms adopted due to the oxidative stress from RONS. As shown in Figs. 4 and 5, *E. coli* and *S.* Enteritidis was more susceptible to the air- and O_2_-based plasma treatments compared to *B. subtilis*. This phenomenon might be connected with the differences in structures, chemical compositions, and molecular organisations of cell walls between gram-positive and gram-negative bacteria^27^. Gram-positive bacteria might be resistant to atmospheric cold plasma treatment because of a thicker outer membrane which may reduce the diffusion of reactive plasma species through the bacterial cell wall^1^. In the case of the chosen models, the inactivation rates for the tested bacteria was comparable during the first inactivation phase, which was evident from *k*_*max1*_- and *D*_*1*_-values (Table 1).

On the other hand, using N_2_-based plasma led to another inactivation behaviours of *E. coli* and *S.* Enteritidis; the inactivation curves showed linearity (Fig. 6). Despite of an absence of resistant fraction, *S.* Enteritidis was more resistant with low inactivation rates compared with *E. coli* and *B. subtilis*. Hertwig et al.^5^ also reported a lower inactivation effect of nitrogen plasma treatment on *Salmonella* Enteritidis PT 30. This might be related to the resistance of *Salmonella* spp. to UV treatment^30^. Obtained OES and FTIR spectra (Fig. 2A and B) showed that N_2_ based plasma emits UV radiation, which could play a role.

## Conclusion

The presented study showed that the MSDBD plasma treatment effectively inactivated food spoilage pathogens, including *E. coli*, *S.* Enteritidis, and *B. subtilis*. By changing the working gases, it was possible to generate plasmas of various compositions with different levels of inactivation. Among the used working gases, ambient air plasma was the most efficient in inactivating the mentioned pathogens. The reactive oxygen and nitrogen species (RONS) generated in plasma are the main agents responsible for the decontamination effect in the case of ambient air-based plasma treatment. Moreover, the inactivation might be related to ozone, whose production efficiency with MSDBD plasma is substantially higher than with ordinary DBD geometries (volume, surface, or coplanar). In addition, water in the form of atmospheric humidity caused the formation of hydroxyl radicals, one of the most potent ROS, and HNO_2_ and HNO_3_ from nitrogen oxides.

According to the results of this study, MSDBD plasma generated in different working gases is a potential technology for inactivating pathogens like *E. coli*, *S.* Enteritidis, and *B. subtilis*. To determine the appropriate treatment conditions for the most effective inactivation, modelling of inactivation kinetics is valuable for helping understand the behaviour of tested microorganisms under various plasma treatments. The biphasic model provided suitable parameters *k*_*max1*_ and *k*_*max2*_, as well as f, which have practical significance and, ultimately confirmed the presence of two subpopulations of tested microorganisms with different sensitivities to plasma treatment. However, since those are preliminary results, more research is required, especially concerning the microorganism—food product—plasma interactions.

## Data Availability

The data that support the findings of this study are available from the corresponding author, [S.M.], upon reasonable request.
